# Age, COVID-19-like symptoms and SARS-CoV-2 seropositivity profiles after the first wave of the pandemic in France

**DOI:** 10.1007/s15010-021-01731-5

**Published:** 2021-11-25

**Authors:** Fabrice Carrat, Nathanael Lapidus, Laetitia Ninove, Hélène Blanché, Delphine Rahib, Paola Mariela Saba Villarroel, Mathilde Touvier, Gianluca Severi, Marie Zins, Jean-François Deleuze, Xavier de Lamballerie, for the SAPRIS-SERO study group, Fabrice Carrat, Pierre-Yves Ancel, Marie-Aline Charles, Gianluca Severi, Mathilde Touvier, Marie Zins, Nathalie Bajos, Florence Jusot, Claude Martin, Laurence Meyer, Ariane Pailhé, Alexandra Rouquette, Alexis Spire, Sofiane Kab, Adeline Renuy, Stephane Le-Got, Celine Ribet, Emmanuel Wiernik, Marcel Goldberg, Fanny Artaud, Pascale Gerbouin-Rérolle, Mélody Enguix, Camille Laplanche, Roselyn Gomes-Rima, Lyan Hoang, Emmanuelle Correia, Alpha Amadou Barry, Nadège Senina, Julien Allegre, Fabien Szabo de Edelenyi, Nathalie Druesne-Pecollo, Younes Esseddik, Serge Hercberg, Valérie Benhammou, Anass Ritmi, Laetitia Marchand, Cecile Zaros, Elodie Lordmi, Adriana Candea, Sophie de Visme, Thierry Simeon, Xavier Thierry, Bertrand Geay, Marie-Noelle Dufourg, Karen Milcent, Delphine Rahib, Nathalie Lydie, Clovis Lusivika-Nzinga, Gregory Pannetier, Nathanael Lapidus, Isabelle Goderel, Céline Dorival, Jérôme Nicol, Cindy Lai, Liza Belhadji, Hélène Esperou, Sandrine Couffin-Cadiergues, Jean-Marie Gagliolo, Hélène Blanché, Jean-Marc Sébaoun, Jean-Christophe Beaudoin, Laetitia Gressin, Valérie Morel, Ouissam Ouili, Jean-François Deleuze, Laetitia Ninove, Stéphane Priet, Paola Mariela Saba Villarroel, Toscane Fourié, Souand Mohamed Ali, Abdenour Amroun, Morgan Seston, Nazli Ayhan, Boris Pastorino, Xavier de Lamballerie

**Affiliations:** 1 Sorbonne Université, Inserm, Institut Pierre-Louis d’Épidémiologie et de Santé Publique, Département de Santé Publique, APHP.Sorbonne Université, 27 rue Chaligny, 75571 Paris Cedex 12, France; 2 Unité des Virus Émergents, UVE, Aix Marseille Univ, IRD 190, INSERM 1207, IHU Méditerranée Infection, 13005 Marseille, France; 3 Fondation Jean Dausset-CEPH (Centre d’Etude du Polymorphisme Humain), CEPH-Biobank, Paris, France; 4 Santé Publique France, Saint-Maurice, France; 5 Sorbonne Paris Nord University, Inserm U1153, Inrae U1125, Cnam, Nutritional Epidemiology Research Team (EREN), Epidemiology and Statistics Research Center-University of Paris (CRESS), Bobigny, France; 6 CESP UMR1018, Université Paris-Saclay, UVSQ, Inserm, Gustave Roussy, Villejuif, France; 7 Department of Statistics, Computer Science and Applications, University of Florence, Florence, Italy; 8 Paris University, Paris, France; 9 Université Paris-Saclay, Université de Paris, UVSQ, Inserm UMS 11, Villejuif, France

**Keywords:** SARS-CoV-2, COVID-19, General population, Cohort, Serologic profile, Neutralizing antibodies

## Abstract

**Background:**

The interplay between age and symptoms intensity on antibody response to SARS-CoV-2 infection has not been studied in a general population setting.

**Methods:**

We explored the serologic profile of anti-SARS-CoV-2 antibodies after the first wave of the pandemic, by assessing IgG against the spike protein (ELISA-S), IgG against the nucleocapsid protein (ELISA-NP) and neutralizing antibodies (SN) in 82,126 adults from a French population-based multi-cohort study.

**Results:**

ELISA-S positivity was increased in 30- to 49-year-old adults (8.5%) compared to other age groups (5.6% in 20- to 29-year-olds, 2.8% in ≥ 50-year-olds). In the 3681 ELISA-S positive participants, ELISA-NP and SN positivity exhibited a U-shaped relationship with age, with a lower rate in 30- to 49-year-old adults, and was strongly associated with COVID-19-like symptoms.

**Conclusion:**

Our study confirms the independent role of age and symptoms on the serologic profile of anti-SARS-CoV-2 antibodies, but the non-linear relationship with age deserves further investigation.

**Supplementary Information:**

The online version contains supplementary material available at 10.1007/s15010-021-01731-5.

## Introduction

Since the emergence of SARS-CoV-2 in China in December 2019 and its spread worldwide, numerous studies have focused on determining the antibody status of the population at different times during the course of the pandemic. Consistent associations between seropositivity and young age, low socio-economic factors and high population density have been reported [1]. However, very few studies have simultaneously explored the response profiles to different anti-SARS-CoV-2 antibodies in the general population using different serological methods, to determine the relationship between these profiles and individual characteristics or symptoms.

In France, SARS-CoV-2-positive RT-PCR tests were first reported on January 24, 2020 and an estimated 4–5% of the adult population in metropolitan France had developed antibodies against SARS-CoV-2 by May 2020 at the end of the first wave of the pandemic [2]. Our objective was to characterize the serological profile to different anti-SARS-CoV-2 antibodies in the general population in France after the first wave of the pandemic, to assess its dynamic over time as well as its association with the participants’ characteristics and their past symptoms.

## Methods

We used data from the SAPRIS (“SAnté, Perception, pratiques, Relations et Inégalités Sociales en population générale pendant la crise COVID-19”)—SERO survey in France. The study has been described elsewhere [2]. It is based on a consortium of prospective cohort studies in the general population including 279,478 adult volunteers with regular access to electronic (internet) questionnaires. Two self-administered questionnaires covering the lockdown and the post-lockdown periods were sent as of April 1, 2020 and returned before May 27, 2020. The questionnaires included socio-demographics, household size and composition, history of COVID-19 diagnosis and SARS-CoV-2 RT-PCR testing, a detailed description of the participant’s symptoms in the previous weeks, and an invitation to perform a serology by self-sampling dried-blood spot (DBS). Participants living in mainland France who completed the questionnaires and who agreed to the serology received a DBS kit to be returned to the centralized biobank after capillary blood collection (CEPH Biobank, Paris, France). Two groups of kits were sent out: the first was a random sample of participants in 3 of the 12 mainland French regions, the second was extended to include all regions of France and all consenting participants. The Elisa test (Euroimmun^®^, Lübeck, Germany) was used to detect anti-SARS-CoV-2 antibodies (IgG) directed against the S1 domain of the spike protein of the virus (ELISA-S). In accordance with the manufacturer’s instructions, a test was considered to be ELISA-S-positive with an optical density ratio ≥ 1.1, ELISA-S indeterminate between 0.8 and 1.1, and ELISA-S-negative, < 0.8. All samples with an ELISA-S test ≥ 0.7 were also tested with an ELISA test to detect IgG antibodies against the SARS-CoV-2 nucleocapsid protein (Euroimmun^®^, Lübeck, Germany, ELISA-NP) using the same thresholds as above and with an in-house micro-neutralization assay to detect neutralizing anti-SARS-CoV-2 antibodies (SN), as described elsewhere with a positive SN defined as a titer ≥ 40 [3]. The sensitivity and specificity of the ELISA-S test at the 1.1 threshold (considering indeterminate results as negative) were reported to be 87% and 97.5%, respectively [4]. Positive/negative concordance of the ELISA-S between DBS and serum was 100%/99.3% [5]. More details on serological methods can be found in [2].

Ethical approval and written or electronic informed consent were obtained from each participant before enrollment in the original cohort. The SAPRIS-SERO study was approved by the Sud-Mediterranée III ethics committee (approval #20.04.22.74247) and electronic informed consent was obtained from all participants for DBS testing. The study was registered (#NCT04392388).

For the present analysis, we selected all participants aged 20 years and older who were invited to provide a serology and returned the DBS before October 1, 2020 with interpretable ELISA-S serologic results.

Seropositivity was evaluated in relation to symptoms reported in the two weeks before completion of the questionnaires and in symptoms that were reported after March 1, 2020 to limit the risk of misclassification with illnesses caused by other seasonal respiratory pathogens. COVID-19-like symptoms (CLS) were defined according to the European Centre for Disease Prevention and Control as at least one of the following: cough, fever, dyspnea, and sudden anosmia, ageusia or dysgeusia [6]. Participants who did not report any of these symptoms on either questionnaire, did not have a positive COVID-19 diagnosis, or who had not experienced cough or fever since the beginning of the year were classified as "No symptoms reported". Other collected symptoms included headaches, rhinorrhea, fatigue, stiffness, myalgia, nausea, diarrhea, chest pain, skin lesions. We used the 2016 French regional population census data to derive post-stratified nationwide age- and gender-adjusted estimates of seropositivity in mainland France, with confidence intervals computed by bootstrapping.

We used logistic regression with stratification in the source cohort to identify factors associated with a positive ELISA-S (versus negative or indeterminate), or factors associated with a positive ELISA-NP (versus negative or indeterminate) or a positive SN (versus negative) in ELISA-S positive participants. Multivariable estimates were adjusted for region, month sampling, age group, gender, household size and living with at least one child.

## Results

A total of 93,610 adult participants were invited to perform the serology between May 4, 2020 and July 29, 2020, 86,913 (93%) returned a DBS before October 1, 2020, and a serology could be performed in 82,126 (88%) participants who were selected for this analysis.

The median age of participants was 60 years (InterQuartile Range, IQR 47–71), 65% were women and the number of available samples varied between 2800 and 15,185 in the 12 different regions of mainland France.

Three thousand six hundred and eighty one participants (4.5%) had a positive ELISA-S, 1323 (1.6%) an indeterminate ELISA-S and 77,122 (93.9%) a negative ELISA-S. With regional post-stratification for age and gender, 5.1% [95% Confidence Interval (95% CI) 4.9%, 5.4%] had a positive ELISA-S, 1.6% (95% CI 1.5%, 1.7%) an indeterminate ELISA-S and 93.3% (95% CI 93.0%, 93.5%) a negative ELISA-S.

**Fig. 1:**
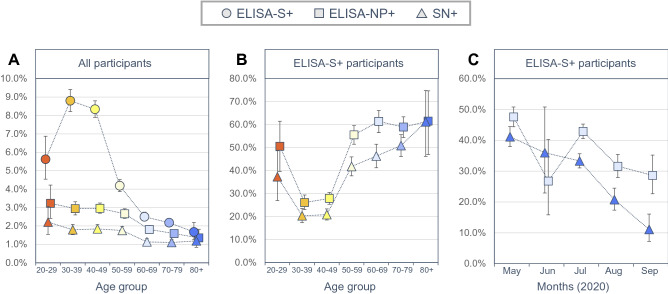
**A** Proportion of participants with positive ELISA IgG against the SARS-CoV-2 spike protein (ELISA-S—circle), with positive ELISA IgG against the SARS-CoV-2 nucleocapsid protein (ELISA-NP—square) and with positive neutralizing anti-SARS-CoV-2 antibodies test (SN—triangle), by age—France, May–September 2020; **B** Proportion of positive ELISA-NP and positive SN in ELISA-S-positive participants, by age; **C** Proportion of positive ELISA-NP and positive SN in ELISA-S-positive participants, by sampling months. Bars represent confidence intervals for proportions

We found a strong association between ELISA-S and age with increased positivity in 30- to 49-year-old adults [8.5% (95% CI 8.2%, 8.9%)] compared to younger [5.6% (95% CI 4.5%, 6.9%)] or older adults [2.8% (95% CI 2.6%, 2.9%)] (Fig. 1A). ELISA-S positivity decreased with the sampling months or in active or ex-smoker (compared to non-smoker), and was higher in women than in men, when living with at least one child, in participants with a higher level of education and in healthcare worker (Supplementary Table 1).

A positive ELISA-NP was found in 1839 (2.3%) and a positive SN in 1200 (1.5%) participants, and it was lower with increasing age (Fig. 1A). In ELISA-S positive participants, 142 ELISA-NP and 304 SN results were not interpretable or missing, 1479 (42%) had a positive ELISA-NP and 1085 (32%) a positive SN. There was a strong association between positivity and age, with a U-shaped pattern, and lower proportions of positive ELISA-NP or SN in 30- to 49-year-old adults (< 30%) compared to younger or older adults (> 40%, Fig. 1B, Supplementary Table 2). Positive ELISA-NP or SN in ELISA-S positive participants was also related to the month of sampling showing in particular a rapid decrease of SN. It was 48% (95% CI 44%, 51%) with ELISA-NP and 41% (95% CI 38%, 44%) with SN in May and 29% (95% CI 23%, 35%) and 11% (95% CI 7%, 16%) in September, respectively (Fig. 1C).

**Fig. 2:**
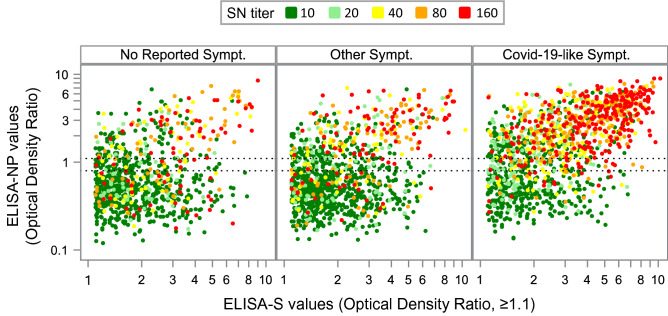
Scatter plot of quantitative serologic results in participants with positive ELISA IgG against the SARS-CoV-2 spike protein (ELISA-S) according to symptoms reported during the first wave of the SARS-CoV-2 pandemic. Each dot represents a triple measurement of ELISA-S, ELISA IgG against the SARS-CoV-2 nucleocapsid protein (ELISA-NP) and neutralizing anti-SARS-CoV-2 antibodies (SN) in one participant with the dot color indicating the SN titer value. Dashed horizontal lines represent threshold limits for positive (1.1) and intermediate (0.8) results as per the manufacturer for ELISA-NP

A total of 1450 ELISA-S-positive participants (39%) reported CLS a median of 3.9 months (Q1 2.1, Q3 4.3) before DBS, 63% were ELISA-NP positive and 49% were SN-positive while 1234 (34%) reported other symptoms, 28% ELISA-NP-positive, 21% SN-positive and 997 (27%) had "No symptoms reported", 25% ELISA-NP-positive and 21% SN-positive (Fig. 2, *P* < 0.001). The association with prior symptoms was independent of age and sampling month.

## Discussion

Based on data from a large general population multi-cohort study, ELISA-S positivity was found to be increased in 30- to 49-year-old adults compared to other age groups. In ELISA-S-positive participants, positive ELISA-NP and SN results decreased over time, in 30- to 49-year-old adults and were strongly associated with COVID-19-like symptoms.

The association of SARS-CoV-2 seropositivity in younger adults has been reported in numerous studies [1]. The decrease in seropositivity in older subjects may be explained by fewer social contacts in this age group than in active, younger adults as well as more stringent preventive behaviors to limit exposure to the virus in those at-risk of complications. The lower rate of seropositivity in 20- to 29-year-old adults is more surprising but may be related to decreased susceptibility, a different immune response to infection like that in adolescents [7], or to a decreased risk of infection due to a lower exposure to children or adolescents (8.5%) compared to the 30- to 49-year-old group (60.9%).

Higher levels of neutralizing antibodies have been reported in other studies after documented SARS-CoV-2 infection in middle-aged adults or the elderly [8]. Also, the presence and severity of symptoms in infected persons have been found to be associated with the intensity of the neutralizing response [8, 9] or the NP response [10]. Our results were consistent with these, showing that both age and the experience of COVID-19 symptoms were independently associated with the seropositive profiles. However, the U-shaped relationship with age was not expected and we suspect that different mechanisms might explain the high levels of SN positivity found in 20- to 29-year-old adults compared to those found in adults aged 50 years or older. The decrease in the positive ELISA-S rate was modest over time, indicating that most infected individuals with mild-to-moderate COVID-19 had robust immunoglobulin G antibody responses against the viral spike protein [9]. In contrast, the decrease in SN titers was marked and rapid which confirms the limited longevity of neutralizing antibodies in certain participants [11].

Our study has several limitations. First, no correction was performed for imperfect test accuracy and the sensitivity of the ELISA-S method in relation to RT-PCR-positive infection was quite low, as reported in other studies [4, 12]. The specificity of the ELISA-S was high, but could explain the inverted U relationship between age and positivity to other serological tests if the proportion of participants with a false-positive ELISA-S was higher in the 30–49 age group compared with other age groups. However, we found no argument in the literature for different sensitivity and specificity of the ELISA-S by age. In addition, this inverted U relationship persisted after adjusting on symptoms, gender, month of sampling, exposure to children, which limits the risk of bias in our analysis due to differential misclassification by age. The second limitation was that only one sample was analyzed per participant, preventing interpretation of the within participant dynamics of the serological response. However, there were obvious trends and multivariable adjustments were performed to control for the major confounding factors associated with decreasing seropositivity over time.

Our study has numerous strengths. Participants were recruited from the general population, preventing bias in the analysis of associations with symptoms that may occur when subjects are recruited during a medical visit or after a hospitalization. Although the date of infection is not known with certainty, positive participants were almost likely infected between February and May 2020, when no variant of interest was circulating in France and vaccinations had not begun.

In conclusion, this large multi-cohort study in the general population confirms that the pattern of seropositivity is strongly related to age and symptoms. Neutralizing antibodies decrease rapidly over time but the relationship between this decrease and increased susceptibility to re-infection must be determined. The non-linear relationship with age deserves further investigation.

## Supplementary Information

Below is the link to the electronic supplementary material.

Supplementary file1 (DOCX 53 KB)

## Data Availability

In regards to data availability, data of the study are protected under the protection of health data regulation set by the French National Commission on Informatics and Liberty (Commission Nationale de l’Informatique et des Libertés, CNIL). The data can be available upon reasonable request to the corresponding author (fabrice.carrat@iplesp.upmc.fr), after a consultation with the steering committee of the SAPRIS-SERO study. The French law forbids us to provide free access to SAPRIS-SERO data; access could however be given by the steering committee after legal verification of the use of the data. Please, feel free to come back to us should you have any additional question.
