# Supplementary material for: Age, COVID-19-like symptoms and SARS-CoV-2 seropositivity profiles after the first wave of the pandemic in France
Source: Infection. 2021 Nov 25;50(1):257–62. doi: 10.1007/s15010-021-01731-5 (PMC8614216; doi:10.1007/s15010-021-01731-5)
Supplement: Supplementary file 1 — Supplementary file1 (DOCX 53 KB) [file 15010_2021_1731_MOESM1_ESM.docx]

Supplementary material

Table 1. Factors associated with a positive ELISA-S (vs negative or indeterminate)

|  | Positive/Total  3681/82126 | % | Odds-Ratio^a^ | 95%CI | Adjusted Odds-Ratio^b^ | 95%CI |
| --- | --- | --- | --- | --- | --- | --- |
| Regions  Haut-de-France  Normandie  Ile de France  Bretagne  Grand Est  Bourgogne France Comté  Centre val de Loire  Auvergne Rhôme Alpes  Pays de Loire  Occitanie  Nouvelle Aquitaine  PACA | 199/4907  94/2800  1097/15185  166/5889  529/7244  121/2821  200/5051  397/9647  164/4629  219/8226  338/10664  157/5063 | 4.1  3.4  7.2  2.8  7.3  4.3  4.0  4.1  3.5  2.7  3.2  3.1 | 1.55  1.34  2.76  1.02  2.85  1.78  1.47  1.58  1.32  Ref  1.17  1.23 | 1.28 ; 1.89  1.04 ; 1.71  2.38 ; 3.20  0.83 ; 1.25  2.74 ; 3.35  1.41 ; 2.23  1.21 ; 1.79  1.34 ; 1.87  1.07 ; 1.62  0.98 ; 1.39  1.00 ; 1.51 | 1.46  1.26  2.30  1.00  2.42  1.73  1.50  1.46  1.24  1.07  1.23 | 1.20 ; 1.78  0.99 ; 1.62  1.97 ; 2.69  0.81 ; 1.23  2.04 ; 2.87  1.37 ; 2.17  1.24 ; 1.83  1.23 ; 1.73  1.01 ; 1.52  0.89 ; 1.28  1.00 ; 1.52 |
| Month sampling (2020)  May  June  July  August  September | 976/14157  56/1066  1783/44898  640/16235  226/5770 | 6.9  5.3  4.0  3.9  3.9 | Ref  0.75  0.63  0.59  0.61 | 0.57 ; 0.99  0.58 ; 0.68  0.53 ; 0.65  0.53 ; 0.71 | 0.87  0.82  0.74  0.76 | 0.66 ; 1.16  0.75 ; 0.91  0.66 ; 0.84  0.65 ; 0.89 |
| Age group (years)  [20-30[  [30-40[  [40-50[  [50-60[  [60-70[  [70-80[  ≥80 | 89/1583  781/8874  1236/14822  624/14914  416/16650  483/22175  52/3108 | 5.6  8.8  8.3  4.2  2.5  2.2  1.7 | Ref  1.61  1.50  0.71  0.42  0.35  0.27 | 1.28 ; 2.02  1.20 ; 1.87  0.57 ; 0.89  0.33 ; 0.53  0.27 ; 0.45  0.18 ; 0.39 | 1.48  1.40  0.73  0.47  0.39  0.29 | 1.18 ; 1.87  1.12 ; 1.77  0.58 ; 0.92  0.37 ; 0.60  0.30 ; 0.51  0.20 ; 0.42 |
| Gender  Male  Female | 1136/28753  2545/53373 | 4.0  4.8 | Ref  1.31 | 1.21 ; 1.41 | 1.20 | 1.11 ; 1.30 |
| Living Area  Rural  <20,000 inhab.  20-000-100,000 inhab.  >100,000 inhab.  Missing | 612/16927  479/12382  580/12443  1809/36400  3974 | 3.6  3.9  4.7  5.0 | Ref  1.11  1.31  1.36 | 0.98 ; 1.25  1.16 ; 1.47  1.22 ; 1.52 | 1.05  1.09  1.08 | 0.93 ; 1.19  0.97 ; 1.24  0.96 ; 1.22 |
| Household size and composition  Nb adults (inc. participant)  1  2  3+  Nb children (<18yrs)  0  1+  Missing | 678/15637  1194/38651  1809/27817  2217/63318  1464/18787  21 | 4.3  3.1  6.5  3.5  7.8 | Ref  0.74  1.39  Ref  2.06 | 0.67 ; 0.81  1.27 ; 1.53  1.92 ; 2.21 | 0.91  0.92  1.22 | 0.82 ; 1.00  0.82 ; 1.05  1.09 ; 1.37 |
| Total household monthly income  <1000€  1000-1499  1500-1999  2000-2999  3000-3999  >4000  Missing | 50/1046  103/2400  213/5615  540/14670  894/20572  1608/31253  6570 | 4.8  4.3  3.8  3.7  4.4  5.2 | Ref  0.92  0.85  0.86  1.01  1.19 | 0.65 ; 1.30  0.62 ; 1.16  0.64 ; 1.15  0.76 ; 1.36  0.90 ; 1.59 | 1.00  0.91  1.02  1.12  1.23 | 0.70 ; 1.41  0.66 ; 1.25  0.75 ; 1.38  0.83 ; 1.51  0.91 ; 1.66 |
| Educational level  <High-school degree  High-school degree or undergraduate  Graduate degree or doctorate  Missing | 205/8895  1455/35181  1572/30378  7672 | 2.3  4.1  5.2 | Ref  1.91  2.56 | 1.64 ; 2.21  2.21 ; 2.97 | 1.25  1.30 | 1.07 ; 1.46  1.12 ; 1.53 |
| Professional activity before lockdown  Student  Working  Looking for a job  Retired  Not working due to health conditions  No professional activity (housewife or husband)  Missing | 27/402  2432/37294  101/1830  763/35337  23/632  70/1764  4867 | 6.7  6.5  5.5  2.2  3.6  4.0 | 1.04  Ref  0.84  0.32  0.53  0.59 | 0.70 ; 1.54  0.68 ; 1.03  0.29 ; 0.36  0.35 ; 0.81  0.46 ; 0.75 | 1.08  0.89  0.80  0.74  0.93 | 0.71 ; 1.64  0.73 ; 1.10  0.67 ; 0.97  0.49 ; 1.14  0.72 ; 1.19 |
| Professional activity during lockdown  Not working  Stopped working  Working from home, remote working  Partially working from home  Working outside home  Other  Missing | 984/39965  415/6368  1298/19132  231/3785  341/5491  71/1207  6178 | 2.5  6.5  6.8  6.1  6.2  5.9 | 0.37  0.95  Ref  0.89  0.91  0.88 | 0.34 ; 0.41  0.85 ; 1.07  0.77 ; 1.03  0.80 ; 1.02  0.69 ; 1.13 | 0.88  1.03  0.98  1.04  1.03 | 0.77 ; 1.01  0.91 ; 1.16  0.85 ; 1.14  0.92 ; 1.18  0.80 ; 1.32 |
| Socio professional category  Farmer  Craftsmen, traders and business owners  Executives and higher intellectual professions  Intermediate occupations  Employees  Workers  Never worked  Other  Missing | 2/175  88/1904  1850/38708  948/23239  564/12043  53/1548  58/1532  41/890  2087 | 1.1  4.6  4.8  4.1  4.7  3.4  3.8  4.6 | 0.21  0.85  Ref  0.86  0.88  0.64  0.95  0.87 | 0.05 ; 0.86  0.68 ; 1.06  0.80 ; 0.94  0.80 ; 0.97  0.49 ; 0.85  0.72 ; 1.24  0.63 ; 1.19 | 0.28  0.94  1.02  0.93  0.87  1.12  0.95 | 0.07 ; 1.12  0.75 ; 1.18  0.94 ; 1.12  0.84 ; 1.03  0.65 ; 1.15  0.85 ; 1.49  0.69 ; 1.32 |
| Essential job position  Healthcare worker Y vs N  Other essential job Y vs N | 224/2817  407/6968 | 8.0  5.8 | 1.65  1.18 | 1.43 ; 1.90  1.06 ; 1.31 | 1.32  0.95 | 1.14 ; 1.53  0.85 ; 1.06 |
| Smoking status before lockdown  Active smoker  Ex-smoker  Non smoker  Missing | 325/8151  1200/32374  1971/37671  3930 | 4.0  3.7  5.2 | 0.71  0.69  Ref | 0.63 ; 0.80  0.64 ; 0.74 | 0.66  0.87 | 0.59 ; 0.75  0.81 ; 0.94 |
| Alcohol use before lockdown (in g/dy)  <5  [5,10[  [10,20[  [20,30[  ≥30  Missing | 1498/31881  676/14391  707/16814  280/7332  210/5732  5976 | 4.7  4.7  4.2  3.8  3.7 | Ref  0.99  0.90  0.81  0.77 | 0.90 ; 1.08  0.82 ; 0.99  0.71 ; 0.92  0.66 ; 0.89 | 1.03  1.00  0.93  0.89 | 0.93 ; 1.13  0.90 ; 1.10  0.81 ; 1.07  0.76 ; 1.04 |
| BMI (kg/m^2^)  <18.5  [18.5; 25[  [25; 30[ (overweight)  >=30 (obese)  Missing | 125/2516  2148/46565  892/22084  325/7402  3559 | 5.0  4.6  4.0  4.4 | 1.07  Ref  0.89  0.94 | 0.89 ; 1.29  0.83 ; 0.97  0.84 ; 1.06 | 0.98  1.07  1.05 | 0.81 ; 1.18  0.98 ; 1.16  0.93 ; 1.19 |
| Chronic diseases  Yes  No  Don’t know  Missing | 2499/50184  903/26627  24/534  4781 | 5.0  3.4  4.5 | Ref  0.74  0.99 | 0.68 ; 0.80  0.65 ; 1.49 | 0.93  1.12 | 0.86 ; 1.01  0.74 ; 1.70 |
| Chronic diseases (Y vs N)  Asthma, COPD, other respir. diseases  Diabetes  Hypertension  Other cardiovascular diseases  Cancer  Anxiety, depression  Other  Missing | 329/10562  100/4452  245/8842  66/2806  138/4132  94/2146  527/15485  4781 | 3.1  2.2  2.8  2.4  3.3  4.4  3.4 | 0.84  0.69  0.67  0.62  0.74  1.01  0.79 | 0.75 ; 0.95  0.56 ; 0.85  0.59 ; 0.77  0.48 ; 0.79  0.62 ; 0.89  0.82 ; 1.24  0.72 ; 0.87 | 0.93  0.88  1.00  0.96  0.89  0.99  0.94 | 0.82 ; 1.05  0.71 ; 1.09  0.87 ; 1.15  0.75 ; 1.24  0.74 ; 1.06  0.80 ; 1.23  0.85 ; 1.03 |

^a^ With stratification on the source cohort. ^b^ With stratification on the source cohort and adjusted on region, month sampling, age group, gender, household size, number of children

Table 2. Factors associated with a positive ELISA-NP or a positive SN in participants with a positive ELISA-S ≥1.1

|  | Positive NP/ Positive ELISA-S  1479/3595 | % | Adjusted Odds-Ratio^a^ | 95%CI | Positive SN/ Positive ELISA-S 1085/3377 | % | Adjusted Odds-Ratio^a^ | 95%CI |
| --- | --- | --- | --- | --- | --- | --- | --- | --- |
| Regions  Haut-de-France  Normandie  Ile de France  Bretagne  Grand Est  Bourgogne France Comté  Centre val de Loire  Auvergne Rhôme Alpes  Pays de Loire  Occitanie  Nouvelle Aquitaine  PACA | 78/196  30/93  551/1067  43/157  292/521  66/120  58/196  138/383  42/158  66/215  68/332  47/157 | 40  32  52  27  56  55  30  36  27  31  20  30 | 1.49  1.11  2.78  0.95  3.03  2.72  0.91  1.28  0.86  Ref  0.64  0.93 | 0.97 ; 2.31  0.64 ; 1.92  1.96 ; 3.95  0.59 ; 1.54  2.08 ; 4.41  1.66 ; 4.47  0.58 ; 1.43  0.87 ; 1.87  0.53 ; 1.39  0.42 ; 0.99  0.58 ; 1.50 | 57/184  20/88  411/1006  28/152  225/491  44/109  49/189  90/358  33/150  40/192  61/316  27/142 | 31  23  41  18  46  40  26  25  22  21  19  19 | 1.74  1.09  2.62  0.95  2.88  2.29  1.38  1.27  1.08  Ref  0.86  0.84 | 1.06 ; 2.86  0.58 ; 2.06  1.74 ; 3.94  0.54 ; 1.67  1.87 ; 4.42  1.32 ; 3.97  0.83 ; 2.28  0.81 ; 1.98  0.62 ; 1.87  0.53 ; 1.40  0.49 ; 1.53 |
| Month sampling (2020)  May  June  July  August  September | 459/964  15/56  749/1745  195/617  61/213 | 48  27  43  32  29 | Ref  0.53  1.21  0.78  0.67 | 0.27 ; 1.04  0.97 ; 1.49  0.60 ; 1.01  0.46 ; 0.98 | 378/918  18/50  556/1668  109/525  24/216 | 41  36  33  21  11 | Ref  1.16  0.94  0.53  0.25 | 0.61 ; 2.21  0.76 ; 1.18  0.39 ; 0.71  0.15 ; 0.40 |
| Age group (years)  [20-30[  [30-40[  [40-50[  [50-60[  [60-70[  [70-80[  ≥80 | 44/87  199/762  336/1204  335/603  253/412  280/475  32/52 | 51  26  28  56  61  59  62 | Ref  0.32  0.40  1.16  1.36  1.36  1.45 | 0.20 ; 0.52  0.25 ; 0.63  0.72 ; 1.86  0.83 ; 2.23  0.80 ; 2.33  0.65 ; 3.21 | 31/83  143/703  238/1142  240/574  177/382  226/444  30/49 | 37  20  21  42  46  51  61 | Ref  0.39  0.43  1.11  1.21  1.62  2.32 | 0.23 ; 0.64  0.26 ; 0.72  0.67 ; 1.85  0.72 ; 2.04  0.92 ; 2.85  1.01 ; 5.33 |
| Gender  Male  Female | 508/1113  971/2482 | 46  39 | Ref  0.81 | 0.68 ; 0.96 | 348/1057  737/2320 | 33  32 | Ref  1.04 | 0.86 ; 1.25 |
| Household size and composition  Nb adults (inc. participant)  1  2  3+  Nb children (<18yrs)  0  1+ | 284/663  606/1174  589/1758  1068/2171  411/1424 | 43  52  34  49  29 | Ref  1.21  1.26  Ref  0.71 | 0.98 ; 1.50  0.96 ; 1.65  0.55 ; 0.91 | 200/620  470/1094  415/1663  778/2031  307/1346 | 32  43  25  38  23 | Ref  1.37  1.08  Ref  0.96 | 1.09 ; 1.72  0.81 ; 1.45  0.73 ; 1.25 |
| Smoking status before lockdown  Active smoker  Ex-smoker  Non smoker  Missing | 114/317  544/1171  736/1927  180 | 36  46  38 | 1.03  1.07  Ref | 0.78 ; 1.35  0.91 ; 1.27 | 79/309  428/1086  528/1814  168 | 26  39  29 | 0.96  1.29  Ref | 0.71 ; 1.29  1.08 ; 1.54 |
| Alcohol use before lockdown (in g/dy)  <5  [5,10[  [10,20[  [20,30[  ≥30  Missing | 598/1464  250/657  296/695  120/275  87/201  303 | 41  38  43  44  43 | Ref  0.91  0.97  1.18  0.92 | 0.74 ; 1.12  0.79 ; 1.19  0.88 ; 1.59  0.65 ; 1.30 | 442/1363  191/626  224/652  93/262  61/190  284 | 32  31  34  36  32 | Ref  0.95  1.02  1.26  0.88 | 0.76 ; 1.19  0.82 ; 1.27  0.92 ; 1.73  0.61 ; 1.28 |
| BMI (kg/m^2^)  <18.5  [18.5; 25[  [25; 30[ (overweight)  >=30 (obese)  Missing | 41/118  825/2106  391/869  144/312  190 | 35  39  45  46 | 0.80  Ref  1.10  1.28 | 0.52 ; 1.22  0.92 ; 1.32  0.98 ; 1.67 | 38/114  614/1964  292/835  99/296  168 | 33  31  35  33 | 1.11  Ref  1.05  1.04 | 0.72 ; 1.72  0.87 ; 1.27  0.78 ; 1.38 |
| Chronic diseases  Yes  No  Don’t know  Missing | 391/874  969/2450  8/23  248 | 45  40  35 | 0.87  Ref  0.49 | 0.73 ; 1.04  0.19 ; 1.25 | 318/840  698/2295  8/23  219 | 38  30  35 | 1.01  Ref  0.85 | 0.84 ; 1.22  0.33 ; 2.24 |
| Chronic diseases (Y vs N)  Asthma, COPD, other respir. diseases  Diabetes  Hypertension  Other cardiovascular diseases  Cancer  Anxiety, depression  Other  Missing | 163/321  54/99  136/237  32/65  56/129  40/91  229/512  248 | 51  55  57  49  43  44  45 | 1.09  0.75  1.07  0.72  0.67  0.99  0.91 | 0.84 ; 1.42  0.48 ; 1.17  0.79 ; 1.44  0.42 ; 1.24  0.45 ; 0.99  0.62 ; 1.59  0.74 ; 1.13 | 124/307  50/95  113/222  31/61  47/120  32/83  187/492  219 | 40  53  51  51  39  39  38 | 0.96  1.16  1.24  1.22  0.80  1.09  1.05 | 0.73 ; 1.26  0.74 ; 1.83  0.91 ; 1.69  0.70 ; 2.14  0.53 ; 1.21  0.66 ; 1.78  0.84 ; 1.31 |
| Experience of illness between March 1st-June 15  No symptoms reported  Other symptoms  Covid-19 Like Symptoms | 241/980  343/1205  895/1410 | 25  28  63 | Ref  1.49  5.69 | 1.21 ; 1.84  4.64 ; 6.97 | 192/930  241/1124  652/1323 | 21  21  49 | Ref  1.22  3.69 | 0.97 ; 1.54  2.99 ; 4.56 |
| Positive RT-PCR (Y vs N)  Missing | 202/232  37 | 87 | 10.0 | 6.66 ; 15.1 | 138/216  33 | 64 | 3.53 | 2.58 ; 4.82 |
| Household contact tested positive (Y vs N) | 133/170 | 78 | 3.84 | 2.58 ; 5.72 | 107/160 | 67 | 3.22 | 2.23 ; 4.64 |

^a^With stratification on the source cohort and adjusted on region, month sampling, age group, gender, household size, number of children
